# The Digestive Tract of Cephalopods: Toward Non-invasive *In vivo* Monitoring of Its Physiology

**DOI:** 10.3389/fphys.2017.00403

**Published:** 2017-06-19

**Authors:** Giovanna Ponte, Antonio V. Sykes, Gavan M. Cooke, Eduardo Almansa, Paul L. R. Andrews

**Affiliations:** ^1^Department of Biology and Evolution of Marine Organisms, Stazione Zoologica Anton DohrnNaples, Italy; ^2^Association for Cephalopod Research (CephRes)Naples, Italy; ^3^Centro de Ciências do Mar do Algarve (CCMAR), Universidade do AlgarveFaro, Portugal; ^4^Department of Life Sciences, Anglia Ruskin UniversityCambridge, United Kingdom; ^5^Centro Oceanográfico de Canarias, Instituto Español de OceanografíaSanta Cruz de Tenerife, Spain

**Keywords:** cephalopods, digestive tract, Directive 2010/63/EU, feces, food intake, nutrition, ultrasound, welfare assessment

## Abstract

Ensuring the health and welfare of animals in research is paramount, and the normal functioning of the digestive tract is essential for both. Here we critically assess non- or minimally-invasive techniques which may be used to assess a cephalopod's digestive tract functionality to inform health monitoring. We focus on: (i) predatory response as an indication of appetitive drive; (ii) body weight assessment and interpretation of deviations (e.g., digestive gland weight loss is disproportionate to body weight loss in starvation); (iii) oro-anal transit time requiring novel, standardized techniques to facilitate comparative studies of species and diets; (iv) defecation frequency and analysis of fecal color (diet dependent) and composition (parasites, biomarkers, and cytology); (v) digestive tract endoscopy, but passage of the esophagus through the brain is a technical challenge; (vi) high resolution ultrasound that offers the possibility of imaging the morphology of the digestive tract (e.g., food distribution, indigestible residues, obstruction) and recording contractile activity; (vii) needle biopsy (with ultrasound guidance) as a technique for investigating digestive gland biochemistry and pathology without the death of the animal. These techniques will inform the development of physiologically based assessments of health and the impact of experimental procedures. Although intended for use in the laboratory they are equally applicable to cephalopods in public display and aquaculture.

## Introduction

Interest in cephalopods and their welfare is stimulated by significant policy changes initiated at European Union (EU) level, that are recognized to have an impact beyond Europe (Di Cristina et al., 2015). Such European Policies and Directives have implications for cephalopods in scientific research, aquaculture or public display, and relevance for fisheries and ecology, i.e., Directive 2010/63/EU, EU Common Fisheries Policy, Marine Strategy Framework Directive, the revision of IUCN Red List (review in: ICES, 2014; Di Cristina et al., 2015; Xavier et al., 2015).

Cephalopods are a special case in a “regulation” context since: (i) it is the first time that research on an entire class of invertebrate has been regulated in the European Union (Smith et al., 2013), and (ii) the species are very diverse in terms of ecological, biological, physiological, and behavioral adaptations (e.g., Packard, 1972; Borrelli and Fiorito, 2008; Grasso and Basil, 2009; Kröger et al., 2011; Albertin et al., 2015).

Daily assessment of animal well-being is required under Directive 2010/63/EU (Fiorito et al., 2015), but few indicators of digestive system physiology are available. Fiorito and co-workers list five (out of 27) signs potentially linked to the digestive system that may indicate an alteration in normal behavior or physiology (see Table 5 in Fiorito et al., 2015).

How can the functionality of the digestive tract be assessed routinely at the “tank–side”?

Here we review and suggest a series of non-invasive and/or minimally invasive approaches (Table 1).

**Table 1:** Summary of parameters that could be used to monitor cephalopod digestive tract functioning by non-invasive or minimally invasive techniques to provide either a direct or indirect insight into the physiology of the digestive tract. Examples are taken mainly from studies on S. officinalis or Nautilus pompilius and/or octopus (mostly O. vulgaris), but all techniques appear equally applicable to squid.

**Parameter monitored**	**Technique**	**Hypothesized potential welfare significance in relation to digestive tract function**	**References**
Body weight	Regular weighing (preferably in water)	Indicates normal food intake, digestive tract functionality, and metabolism	Nixon, 1966; Joll, 1977; Boyle and Knobloch, 1982; Domingues et al., 2002; Semmens et al., 2004; García-Garrido et al., 2010; Sykes et al., 2014
Predatory behavior and interest in food	Behavioral observations	Normal appetitive drive, major digestive tract pathology, unlikely	Oestmann et al., 1997; Borrelli and Fiorito, 2008; Amodio et al., 2014
	Food seeking behaviors, time to capture live or alternative prey		
Food intake (amount, normal time course of ingestion, and feeding frequency)	Behavioral observations (including video recording)	Ingestion mechanisms functional, major digestive tract pathology, unlikely	Guerra and Nixon, 1987; Grisley et al., 1999; Semmens et al., 2004; García-Garrido et al., 2010; Lamarre et al., 2012
	Inspection of tank for uneaten food and residues	Visceral pain (if present in cephalopods), unlikely	
Oro-anal transit time	Dye, indigestible, or radio-opaque markers in the food	Normal digestive tract functionality	Bidder, 1957; Westermann et al., 2002
Presence of food in the digestive tract, normally distributed and contractile activity present	Ultrasound, X-ray (±contrast medium)	Indicates ingestion of food and, if normally distributed, shows lack of structural defects and indirectly normal motility (visible with ultrasound, see Figure 1)	Westermann et al., 2002; Figure 1 (ultrasound), *this paper*
	Direct observation in transparent paralarvae		
Appearance of digestive tract mucosa	Endoscopy (digestive tract)	Identification of mucosa defects and presence of parasites in the lumen	No publications in cephalopods (but see Sykes et al., 2017)
			Utilized in finfish Moccia et al., 1984, crabs Heinzel et al., 1993, and veterinary medicine Sladakovic et al., 2017
External appearance of rectum	Endoscopy (mantle)	Cysts of some parasites (e.g., *Aggregata* ssp) may be visible	No publications in cephalopods
			For *Aggregata* see Mayo-Hernández et al., 2013
Digestive gland size (% body weight) and density	High resolution ultrasound	Global indicator of metabolic status and functionality of digestive tract	García-Garrido et al., 2010; Speers-Roesch et al., 2016
		Increased water content indicates catabolism and should be detectable during sonographic examination	Supplementary Figure 1, *this paper*
Digestive gland biochemistry	Needle biopsy	Indicator of overall metabolic status and normal functioning of the digestive tract^a^	No publications on needle biopsy in cephalopods, but well established in humans (e.g., Kim and Shin, 2017)
			For biochemistry approaches see: Lopes et al., 2014; Lamarre et al., 2016; Speers-Roesch et al., 2016; Penicaud et al., 2017
Mantle muscle thickness and gill morphology (remodeling)	Ultrasound	May indicate severe food deprivation or chronic failure of digestive tract function	No formal studies in cephalopods, but for mantle/gill morphology during food deprivation see Lamarre et al., 2012 See Figure 1, *this paper*
Regurgitation of food	Behavioral observation (including video recording)	May indicate toxic food, digestive tract obstruction, or disordered neural control	Unpublished observations cited in Andrews et al. (2013)
			A. Sykes and E. Almansa, unpublished observations
Defecation frequency change	Behavioral observation (including video recording)	Indirect measure of digestive tract functionality, but only reflects handling of previous meal	No formal studies in cephalopods
		May indicate stress, digestive tract infection, or alteration in secretion, absorption of motility, defective control	
Fecal form, color, composition change	Behavioral observation (including video recording)	Form may reflect motility of the lower digestive tract; color most likely related to diet; composition reflects epithelial water transport, but parasites/bacteria may be present and cytology may eventually reveal disease	No formal studies (see main text for incidental observations in the literature)
	Fecal collection (molecular and/or cytological analysis)		
Urine composition: Tank water [ammonia]	Chemical or electrochemical detection (potentially real time, continuous monitoring)	Indication of nitrogenous metabolism reflecting protein intake and overall metabolic status	Boucher-Rodoni and Mangold, 1985; Segawa and Hanlon, 1988; Katsanevakis et al., 2005; García et al., 2011
Oxygen consumption	Metabolic chamber	Assessment of normal metabolism and indication of catabolic state of O_2_:N_2_ ratio	Boucher-Rodoni and Mangold, 1988; Valverde and García, 2005; Capaz et al., 2017
Circulating nutrients, metabolites	Hemolymph sampling	Indicates normal functioning of the digestive tract and particularly the digestive gland	Aguila et al., 2007; Linares et al., 2015
Circulating hormones, including those regulating the digestive tract	Hemolymph sampling	Insights into the control of the digestive tract, but also potential larkers of immune response (e.g., TNFα) or stress	No formal studies of digestive tract hormones in cephalopods, but see Zatylny-Gaudin et al., 2016 for peptidome study including haemolymph for candidates
Circulating inflammatory markers	Hemolymph sampling	May indicate the presence of a pathogen in the digestive tract	e.g., Castellanos-Martínez et al., 2014a,b; Gestal and Castellanos-Martínez, 2015

*Obstruction of the hepatopancreatic duct would prevent both water and nutrient absorption by the digestive gland (Wells and Wells, 1989)*.

## Predatory response and food intake

The vast majority of cephalopod species are active predators, and prey attack is considered to be a good indicator of overall health (Fiorito et al., 2014). Latency to attack either a live- or an artificial crab is used as an indication of health in *Octopus vulgaris* (Amodio et al., 2014), and a prompt feeding response to a live fish is proposed for cuttlefish and squid as an indication of full recovery after transport (Oestmann et al., 1997). Latency to attack may vary between individuals (Lee et al., 1991) which probably denotes different temperaments (e.g., Sinn et al., 2001; Sinn and Moltschaniwskyj, 2005; Borrelli, 2007; Carere et al., 2015), and these differences should be taken into account (see discussion in Borrelli and Fiorito, 2008). In addition, it may be influenced by housing, as shown by laboratory reared juvenile cuttlefish that have a shorter attack latency (three times faster) if housed together (Warnke, 1994), and if there is more available space (Boal et al., 1999).

Ingestion of a normal amount of food (assuming “normal” can be defined for species, age, sex, and temperature) at a regular frequency is probably the most useful indicator of health. However, monitoring food intake requires knowledge of the amount of food provided and of possible remains. Therefore, tanks should be inspected for empty carapaces, shells, and other residues as well as uneaten food. For prepared diets whenever accepted, allowance needs to be made for portions of the food pellet lost, due to leaching and disaggregation, and not ingested.

If animals are fed live prey, uneaten specimens must be removed to avoid possible welfare issues; the prey may become the “predator,” particularly if the size difference between the two individuals is not large (e.g., a small cuttlefish and a shrimp). In addition, leaving uneaten prey may lead to inaccurate estimation of intake over a particular time period.

An additional issue is to ensure that every animal has sufficient food. This can be challenging for species which may be housed in groups such as *S*. *officinalis, Loligo vulgaris* (or other squid) or *O. vulgaris* that are kept under culture conditions. This may be assisted by observation, but feeding hierarchies should be taken into consideration when animals (e.g., cultured cuttlefish) are housed together (see discussion in Warnke, 1994). To circumvent these issues, and again in an aquaculture context animals are fed *ad libitum*, but accurate monitoring of food remains is required.

Food intake may decrease with increasing size in cephalopods, and depends on food availability, its quality and size, the duration of digestion, maturation, and temperature (Mangold, 1983).

Feeding behavior and food intake may be affected by experimental procedures; resumption of the normal status should be assessed at individual animal level. A few examples from the literature are provided below.

*Octopus tetricus* attacked and ate crabs within 15 min after brief anesthesia used to facilitate handling for weighing (Joll, 1977). Similarly, *S. officinalis* resumed feeding on grass shrimp 7 min after recovery from anesthesia used for handling (Gonçalves et al., 2012). Cuttlefish resumed feeding 48 h post-surgery after kainic acid lesion of the vertical lobe (Graindorge et al., 2008). *O. vulgaris* performed a normal predatory response 1 h after anesthesia and arm surgery (Shaw et al., 2016). However, recovery of the attack response following cold water “anaesthesia” in the same species is prolonged with respect to circumstances when magnesium chloride is used as agent (Agnisola et al., 1996).

Cephalopod paralarvae or other early stages provide further challenges. Ingestion of food and estimation of food intake may be assessed by direct observation or through the use of microfluorospheres (around 10 μm diameter) included in the food (see Villanueva and Norman, 2008, and below), since at early stages most species are transparent.

## Body weight and deviation from “normality”

Loss of body weight or growth below particular pre-set limits are frequently used as humane end points (see discussion in: Smith et al., 2013; Fiorito et al., 2015) in regulated procedures. However, setting limits requires knowledge of the normal variations in body weight for the species studied and the housing conditions (particularly water temperature). Total body length is not considered a valid index of growth in cephalopods because of variables including the elasticity of tentacles and arms, and differences in relative growth linked to sex and season (e.g., Bello, 1991; Cortez et al., 1999; Pierce et al., 1999; Sivashanthini et al., 2009).

Gross growth efficiency is dependent on food quality, its ingestion and water temperature (Mangold, 1983). Growth curves for several cephalopod species in captivity show a reduction in growth rate over Winter, due to a temperature decrease in open aquaria (e.g., Nixon, 1966; Joll, 1977; Boyle and Knobloch, 1982; Domingues et al., 2002). However, in constant temperature aquaria, a reduction in growth rate is most likely due to reduced food intake.

Additionally, the available bottom area of the tank and the stocking density may also influence growth rates (see for example: Forsythe et al., 2002; Correia et al., 2005; Delgado et al., 2010; Domingues and Márquez, 2010; Domingues et al., 2010).

The contribution of total body water (intra- and extracellular) to body weight should not be overlooked. Cephalopods ingest and absorb water from the sea and from the diet via the digestive tract, and prevention of fluid absorption from the digestive tract leads to ~10% loss in body weight in 24 h in *O. vulgaris* (Wells and Wells, 1989, 1993).

Body weight loss should always be investigated, and if a reduction in food intake is identified as the primary cause then this also needs investigation.

Body weight and food intake decrease in several species with maturation (e.g., Mangold and Boletzky, 1973; Lee, 1995) and around the time of egg laying, and since this may affect females, their reproductive status should be checked in cases of evident reduction of body weight/food intake. In females of many, but not all species, reproductive status is accompanied by loss of body weight due to reduced or absent food intake (e.g., Wodinsky, 1977; but see Sykes et al., 2013b), and reduction of food intake may also occur in senescent mature males (Anderson et al., 2002).

Individual tissues may be affected to a greater degree than is apparent from body weight. For example, the proportionate weight loss of the digestive gland in food deprived *O. vulgaris* is greater than might be suspected from the change in body weight (see Supplementary Figure 1).

If an animal is losing weight, but food intake is within normal limits then the cause is likely to be the functioning of the digestive tract. However, as the pathophysiology of the cephalopod digestive tract has not been studied in any detail, we can only hypothesize about the causes by analogy with vertebrates (see Table 1).

Body weight is probably the best overall indicator of adequate nutrition, but it may not be the most practical parameter to use for routine health assessment. Repeated measures of body weight show variability (Nixon, 1971) and may induce effects linked to handling (e.g., Locatello et al., 2013). Indeed, the effects of repeated measures of body weight on animal welfare have not been properly investigated, to the best of our knowledge. Repeated handling of the animal may be stressful (for example see: Malham et al., 1998; Grimaldi et al., 2013). According to Nixon (1966) frequent handling of *O. vulgaris* may reduce growth rate, but this does not appear to be the case in cuttlefish (Sykes et al., 2003, 2013a,b).

Weighing usually requires removal of the animal from its tank for measurement in air, although animals can be weighed in water (Aronson, 1982). Body weight may not be a sufficiently sensitive welfare indicator for daily assessment, although it may be useful over longer intervals. However, for species housed in groups reliable identification of individuals is required, and several methods are described for cephalopods (e.g., Huffard et al., 2008; Ikeda et al., 2009; Zeeh and Wood, 2009; Byrne et al., 2010; Barry et al., 2011; Estefanell et al., 2011; Sykes et al., 2017).

## Oro-anal transit time

This is the time from food ingestion until exit as feces, and provides an overall measure of the key digestive tract functions of motility, secretion, and absorption. The most common method is to mark the food with a chemical marker (e.g., carmine in Bidder, 1957) or to incorporate an indigestible marker into the food (e.g., glass beads encapsulated in *Artemia* nauplii, Villanueva and Norman, 2008). However, if particular dietary constituents are associated with a fecal color change then by diet switching it would be possible to estimate transit time for that food.

**Figure 1:**
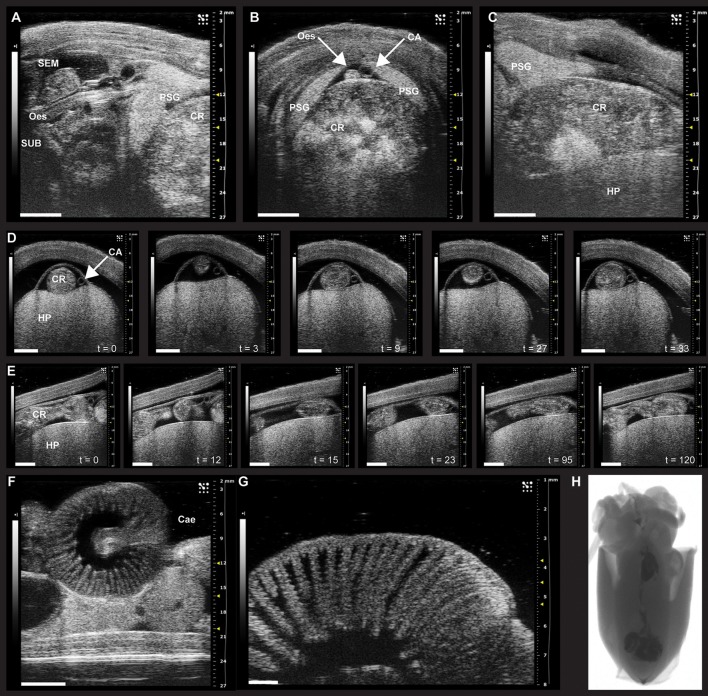
Sonographic scanning of the digestive tract of *Octopus vulgaris* **(A–G)** and X-ray imaging in juvenile *S. officinalis* **(H)**. **(A–G)** The digestive tract of *O. vulgaris* as it appears during ultrasound examination (VEVO 2100, VisualSonics). **(A–C)** The anterior part of the digestive system (note the crop full of food) and its relationship to other parts within the mantle. **(A)** Ultrasound examination in the longitudinal plane with supra- (SEM) and sub-oesophageal masses (SUB, sagittal view) and the esophagus (Oes) and the crop (CR, on the right). The posterior salivary glands (PSG) are also clearly identifiable. **(B)** Sonographic scanning using a transverse plane reveals a distended crop (CR) full of food, the esophagus (Oes), and the cephalic aorta (CA) lying on its dorsal surface between the posterior salivary glands (PSG). **(C)** Sonographic examination (longitudinal plane) showing one posterior salivary gland (PSG) with its typical leaf-shaped appearance, the distended crop (CR), and the hepatopancreas (HP), ventrally. **(D)** A sequence of frames from the sonographic examination (transversal plane) of the octopus digestive tract octopus reveals the crop of an animal fed 6 h before the ultrasound scan (about 30 s); the peristaltic motility of the crop is evident through the sequence of snapshots (from t = 0 to 33 s) with contractions and relaxations moving the crop contents. **(E)** The sequence of frames taken from the same animal during ultrasound examination in the longitudinal plane identifies contraction and relaxation of the crop dividing the bolus. **(F)** The caecum with its characteristic spiral organization as it appears during sonographic scanning. **(G)** High resolution (48 MHz) ultrasound scanning of the caecum showing the “villi-like” structures. **(H)** X-ray imaging of food labeled with contrast medium (barium sulfate) to monitor the course of digestion in juvenile *S. officinalis*. Scanning performed with a Kodak DXS-4000 Pro system on anesthetized individual. CA, cephalic aorta; Cae, caecum; CR, crop; Oes, esophagus; HP, hepatopancreas; PSG, posterior salivary gland; SEM, supra-oesophageal mass; SUB, sub-oesophageal mass. Images provided here resulted from examinations carried out in compliance with local regulations, and for veterinary purposes. Scale bar, **A–F**: 5 mm; **G**: 1 mm.

A study in *Nautilus pompilius* applied X-ray imaging of food labeled with contrast medium (barium sulfate) to monitor the time course of digestion (about 12 h at 18–19°C Westermann et al., 2002). This method has been also explored in *S. officinalis* (Figure 1H). However, this requires brief but repeated anesthesia which, together with restraint and handling, is likely to affect the oro-anal transit time of the animal.

The relatively few studies undertaken measured oro-anal transit times (see Supplementary Table 1) ranging from 2–10 h in squid (temperatures 16–22°C) to 8–24 h *in S. officinalis* (14–23°C), and 8–30 h in octopus (10–30°C). Overall, transit is faster in animals living at higher water temperatures; the slowest time we were able to find was for *Benthoctopus levis* (>30 h at 6°C: Mangold and Lu, unpublished, cited in Mangold and Bidder, 1989).

High resolution ultrasound may provide a method for monitoring movement of digestive tract contents (see below), provided that the animal could be adapted to remain relatively quiescent during repeated imaging sessions lasting a few minutes. This approach is tractable in cuttlefish and octopus, but may be problematic with squid because of their preference to moving in the water column rather than remaining quiet on the bottom of a tank. It is important that measurements are taken in non-sedated and unrestrained animals as this will modify the results.

## Fecal output

Fecal analysis provides a non-invasive method for monitoring digestive tract functionality. Defecation and fecal composition are considered separately.

### Defecation

Based upon transit times (see above) and tank inspection it is usually assumed that defaecation takes place at least once daily in *S. officinalis* and *O. vulgaris*, but this has yet to be confirmed by a direct study.

Defecation is highly likely to be under central nervous system control as “reflex” expulsion of feces, in the presence of a predator when an animal is attempting to hide, would be disadvantageous. Central control of defecation is likely to be via the visceral nerve originating in the palliovisceral lobe (part of the posterior sub-oesophageal mass) which in *O. vulgaris* is described as supplying specific branches with relatively large axons (~5 μm) to the terminal rectum and anal flaps (Young, 1967). Injection of 5-hydroxytryptamine (5-HT) into the brain blood supply (via an implanted cannula in the dorsal aorta) evoked defecation in *O. vulgaris*. This was not induced by nicotine, gamma amino butyric acid, and L-glutamate injections (Andrews et al., 1983). Defecation was not evoked by 5-HT following removal of the supra-oesophageal lobes, suggesting that it may be under “higher” motor control.

Descriptions of defecation in cephalopods are rare; in both cuttlefish and *O. vulgaris* feces are expelled from the anus in a “rope” and are ejected from the mantle cavity via the siphon by mantle contraction referred, for octopus, as a “cough” (Wells, 1978).

Studies of defecatory behavior are needed to establish normal patterns for each laboratory housed species in relation to the diet. In this way criteria can be set for when a change (increase or decrease) requires investigation or intervention.

### Fecal appearance and composition

Descriptions of the appearance of cephalopod feces are scant. In *N. pompilius* fed on shrimp feces are described as “red-brown threads, 2 cm in length and with no solid components” (Westermann et al., 2002, p. 1620). These appear “long filiform, but quite variable in size and in color” in *O. vulgaris* (Taki, 1941), and grayish brown in color when the animals are fed bivalves. An orange brown color is more characteristic of the feces in *O. vulgaris* fed on crabs, and an orange/red color is characteristic of feces of *S. officinalis* fed on grass shrimp or crabs most likely due to carotenes. Feces after feeding animals with fish or prepared diets not rich in crustaceans will lack any obvious pigmentation and may be white. The “fecal ropes” have an obvious mucus coating and presumably contain excretory products of metabolism from the digestive gland and any undigested or unabsorbed food. Feces may contain “chips of cuticle” and fish scales (Wells, 1978) and dead cells (a possible source of cells for genotyping) sloughed from the digestive tract epithelium or the digestive gland, digestive tract flora, and shed parasites and cysts.

Steroid hormones have been detected in the feces of *Enteroctopus dofleini* (Larson and Anderson, 2010), further illustrating the potential of feces as source of biomarkers thus serving as indicators of animals' health and welfare. Overall, the feces are an overlooked potential source of information about the physiology of the digestive tract and their utility as a non-invasive monitor of animal health should be investigated.

## Endoscopy and ultrasound

Endoscopy is a technique widely used for human and veterinary clinical investigation of the digestive tract (for example see Fritscher-Ravens et al., 2014; Sladakovic et al., 2017) to examine the mucosa for abnormalities (e.g., polyps, parasites) or to perform a biopsy for subsequent analysis and for some surgical procedures. The technique has also been used to investigate finfish (Moccia et al., 1984) and crab digestive tract (Heinzel et al., 1993). Endoscopy requires sedation or general anesthesia so the potential stress of this must also be taken into account when considering welfare implications.

The size of endoscopes is a limiting factor in the application to investigate the cephalopod digestive tract with the restriction placed on the esophagus by the supra- and sub-oesophageal lobes and their connecting circum-oesophageal structures being a particular issue. The lower digestive tract is, in theory, accessible to endoscopic inspection via the anal sphincter, but the size of the endoscope will again be a limiting factor. In addition, as far as can be ascertained in all cephalopods, the intestine exits the gastro-caecal junction running caudally and dorsal, but during its course turns rostrally and ventral to exit the mantle near the siphon. Therefore, inspection of the proximal intestine would require a very flexible endoscope.

Ultrasound is utilized for non-invasive imaging of the mantle, vasculature, brain and arms of cephalopods. Ultrasonographic examination can be undertaken without sedation or anesthesia as carried out in *S. officinalis* (King et al., 2005; King and Adamo, 2006) or in *O. vulgaris* (Grimaldi et al., 2007). However, in other circumstances light anesthesia is required to ensure stable images for quantitative analysis of arm or brain morphology (Grimaldi et al., 2007; Margheri et al., 2011).

If an animal stops eating for no apparent reason, ultrasound may help to investigate the digestive tract and search for an obstruction; it should also be possible to view contractile activity of the crop, stomach, caecum or intestine as illustrated in Figure 1. Since the digestive gland decreases in weight, but has an increased % water with increasing duration of food deprivation (see Supplementary Figure 1) it may be possible to use ultrasound measurements of size and density as an index of the metabolic status of the animal, contributing to overall welfare assessment.

As cephalopods experiencing severe food deprivation mobilize lipids from the digestive gland and proteins from muscle and the gills (Lamarre et al., 2012, 2016; Speers-Roesch et al., 2016), ultrasound examination of these structures may provide insights into the overall health status of the animal, particularly if the same structures were imaged on arrival in the laboratory.

## Closing remarks

The assessment of cephalopod digestive tract function through non-invasive methods needs to be developed further but the above overview highlights key areas (Table 1). For example, a specific analysis of the oro-anal transit times will facilitate species comparisons, investigation of the effects of environmental change, assessment of the impact of pathogens, investigation of neural and hormonal control, and provide standardized methods for comparison of experimental diets for use in aquaculture. Deviation of body weight from normality is considered a key welfare indicator, and the impact of prolonged food deprivation on welfare should be taken into account. Analysis of fecal composition will also give insights into absorption and secretion in the digestive tract especially if combined with measurements of metabolites in the haemolymph and/or digestive gland using minimally invasive techniques (Lamarre et al., 2012, 2016; Speers-Roesch et al., 2016). Simple methods for assessment of digestive tract function will also facilitate comparative studies of a wider range of species including those found more frequently in public aquaria.

Although we focused our attention on a series of possible markers of digestive tract function to be monitored through routine assessment at the “tank-side,” daily assessment of health and welfare largely relies on observation of the animal. Understanding the external manifestations, including behavioral changes, of underlying digestive tract pathophysiology will be essential to improve welfare assessment tools for cephalopods.

## Author contributions

All authors contributed to the manuscript and agreed on the final version.

### Conflict of interest statement

The authors declare that the research was conducted in the absence of any commercial or financial relationships that could be construed as a potential conflict of interest.
