# Supplementary material for: The Digestive Tract of Cephalopods: Toward Non-invasive In vivo Monitoring of Its Physiology
Source: Front Physiol. 2017 Jun 19;8:403. doi: 10.3389/fphys.2017.00403 (PMC5474479; doi:10.3389/fphys.2017.00403)
Supplement: Supplementary file 1 [file Presentation1.pdf]

Supplementary Data

The digestive tract of cephalopods:
towards non-invasive *in vivo* monitoring of its physiology

Giovanna Ponte[1,2,✉,*], Antonio V. Sykes[3,*], Gavan M. Cooke[4],
Eduardo Almansa[5], Paul L.R. Andrews[1,2]

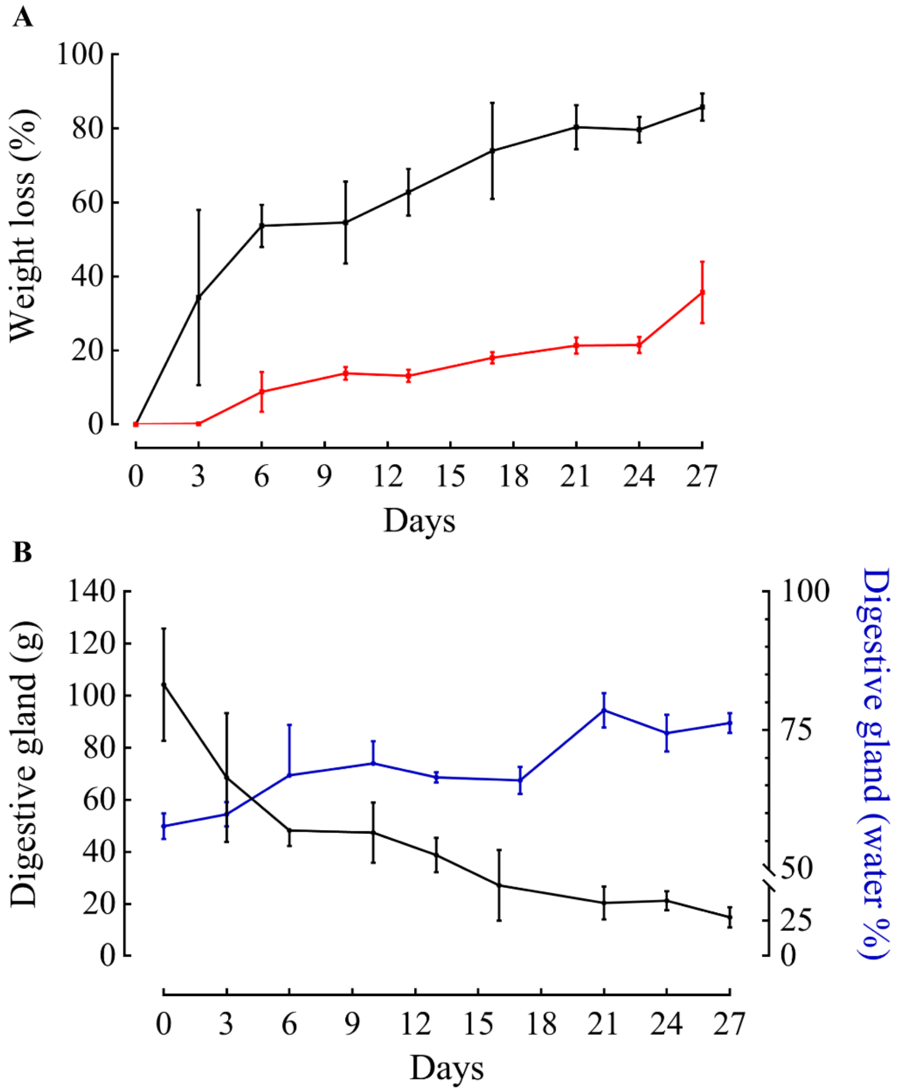

Supplementary Figure 1. Differential response of body weight and digestive gland to weight loss in *Octopus vulgaris* due to prolonged starvation tissues. Data originated from table 1 of García-Garrido et al. (2010) where total body-, digestive gland weight, weight loss, and digestive gland index, of starving *O. vulgaris* for 27 days are presented.

A. Comparison of body weight (**red**) and digestive gland (**black**) weight loss (expressed as %) in *O. vulgaris* deprived of food during the 27 days of starvation experiment. (Day 0, body weight = 1618.3 ± 175.5 g, N = 30; digestive gland = 104.3 ± 21.6 g, N = 3). **B.** Digestive gland weight (**black**, left axis) and % water content in the digestive gland (**blue**, right axis) changes during days in *O. vulgaris* during food deprivation. Values are presented as mean ± SD (N=3 for each data point).

These data indicate that the proportionate weight loss of the digestive gland in food deprived *O. vulgaris* is greater than might be suspected from the change in body weight. Since the digestive gland decreases in weight, but has an increased % water with increasing duration of food deprivation, we suggest it may be possible to use ultrasound measurements of size and density as an index of the metabolic status of the animal, contributing to overall welfare assessment.

Supplementary Table 1. Total times for digestion/oro-anal transit in several species of cephalopods. The data derived mostly from juvenile and adult animals, faster transit times are likely to occur in the paralarvae/hatchling stages. The duration given in some cases (*) is the time taken from ingestion to the anal exit of the marker or food (i.e. the oro-anal transit time) whereas in other studies, particularly those where the distribution of digestive tract contents is analysed *post mortem*, the expression "duration of digestion" is used which is taken as the time when digesta appear in the terminal intestine/rectum. This latter time will be shorter than oro-anal transit time, as residence time in the terminal intestine, pre-defaecation, will not be included.

This table illustrates the paucity of recent data using more modern marker techniques. For example, chromium oxide and yttrium oxide have been validated for use in digestibility studies in cephalopods. In *S. officinalis* the faeces appear greenish in colour when chromium oxide (Cr_2O_3) is used. After yttrium oxide (Y_2O_3) the faeces do not show the greenish colour. These methods may be utilized to enable direct comparison of oro-anal transit times between species or diets using non-invasive techniques. Although data on oro-anal transit times is scant there is data from individual species on the time food spends in various regions of the digestive tract although again this involves *post mortem* analysis of digestive tract contents at fixed times after.

Species	Life stage[1]	Duration	Temperature	Technique/marker	Reference	Comment
Nautilus pompilius	Juvenile	12h*	18-19°C	Barium sulphate labelled shrimps (*Crangon crangon*) plus X-ray and computational tomography	Westermann et al. (2002)	A rare example of a study using a non-invasive technique to measure total transit without killing the animals to measure contents at various time points
Sepia officinalis		18-24h	14-15°C	Carmine dye injected into crabs (40-50 mm carapace width)	Bidder (1957)	Preliminary observations reported in a paper primarily on *O.vulgaris*
		20h	15°C	Dye (Nile blue or Neutral red) injected into the food	Boucaud-Camou and Pequignat (1973)	Fed
		15h	20°C			
	Adult	8h?	18-19°C	Laurell Fused Rocket Immunoelectrophoresis technique on stomach, caecum, digestive gland and intestine	Kear and Boyle (1992)	Fed krill slurry (*Euphausia suberba*). Digestive gland ideal for serological studies
	Juvenile and Adult	24h	15.5°C	weighing the stomach full and emptied	Quintela and Andrade (2002)	Fed shrimp (*Palaemonetes varians*)
		21h	18°C			
		10h	23°C			
Sepioteuthis lessoniana	Hatchling Juvenile	2-4h	22-27°C	weighing the stomach and caecal sac full and emptied	Segawa (1993)	Fed atherinid fish (*Atherion elymus*, *Iso flosmaris*) and mysid (*Siriella longipes*)
	Juvenile	4h	24-27°C	histology contrasting fed and unfed individuals	Semmens (2002)	Fed juvenile mullet

Species	Life stage[1]	Duration	Temperature	Technique/marker	Reference	Comment
Loligo vulgaris	Juvenile	4.5-6h	18°C	Iron saccharate and finely ground-carmine, and with nile blue (sulphate) embedded in agar-agar jelly or gelatine jelly inserted into the food (fish)	Bidder (1950)	
	Adult	8-10h	16°C	Weighing the stomach and caecal sac full and emptied	Lipiński (1987)	Fed live mullet (*Mugil* sp.)
Loligo opalescens	Juvenile	7h	18-19°C	Weighing the stomach full and emptied	Karpov and Cailliet (1978)	Squid may slow their digestion during night hours, thus having small amounts of food in their stomachs throughout the night hours
Illex illecebrosus		12h	10-11°C		Rowe and Mangold (1975)	
Octopus vulgaris	Juvenile/Adult?	16h	14°C	Crab (*Carcinus maenas*) labelled with Indian ink plus direct observation	Boucaud-Camou et al. (1976)	
	Juvenile and Adult	12h	18-19°C	Weighing the different regions of digestive tract full and emptied	Boucher-Rodoni and Mangold (1977)	Time affected by sex and sexual maturation - faster in immature than mature males and in males than females
	Young (VML 75-100mm)	14-15h	23°C	Carmine dye injected into crabs (*Maja* sp., 40-50mm carapace width)	Bidder (1957)	Time given is for "end or nearly end" of digestion; whole gut reported ot empty by 18h
Octopus cyanea	Juvenile and Adult	12h	30°C	Weighing the different regions of digestive tract full and emptied. Fed crab (*Cardisoma carnifex*)	Boucher-Rodoni (1973)	Time given is the time at which the gut was empty after the beginning of the meal
Octopus maya	Juvenile	8h	26±2°C	Histology contrasting fed and unfed individuals	Martínez et al. (2011a,b)	Fed semi-moist crab paste (95%) mixed with natural gelatin as agglutinant (5%)
Muusoctopus levis		> 30h	6°C		Mangold and Lu, unpublished, cited in Mangold and Bidder (1989)	Fragments of food present in the crop 30h after capture
Eledone cirrhosa		30h	10°C		Boucher-Rodoni (1976)	
		20h	15°C			
		15h	20°C			

[1] Reported as included in the original studies.

References

Bidder, A. (1957). Evidence for an absorptive function in the liver of *Octopus vulgaris* Lam. *Pubblicazioni Stazione Zoologica Napoli* 29, 139-150.

Bidder, A.M. (1950). The digestive mechanism of the European squids *Loligo vulgaris*, *Loligo forbesii*, *Alloteuthis media* and *Alloteutihis subulata*. *Journal of Cell Science* 3, 1-43.

Boucaud-Camou, E., and Pequignat, E. (1973). Etude expérimentale de l'absorption digestive chez *Sepia officinalis* L. *Forma et functio* 6, 93-112.

Boucaud-Camou, E., Boucher-Rodoni, R., and Mangold, K. (1976). Digestive absorption in *Octopus vulgaris* (Cephalopoda: Octopoda). *Journal of Zoology* 179, 261-271.

Boucher-Rodoni, R. (1973). Vitesse de digestion d'*Octopus cyanea* (Cephalopoda: Octopoda). *Marine Biology* 18, 237-242.

Boucher-Rodoni, R. (1976). Etude histologique du tube digestif de deux Cephalopodes, *Eledone cirrosa* (Octopoda) et *Illex illecebrosus* (Teuthoidea) au cours de la digestion. *Cahiers de Biologie Marine*.

Boucher-Rodoni, R., and Mangold, K. (1977). Experimental study of digestion in *Octopus vulgaris* (Cephalopoda: Octopoda). *Journal of Zoology* 183, 505-515.

García-Garrido, S., Hachero-Cruzado, I., Garrido, D., Rosas, C., and Domingues, P. (2010). Lipid composition of the mantle and digestive gland of *Octopus vulgaris* juveniles (Cuvier, 1797) exposed to prolonged starvation. *Aquaculture International* 18, 1223-1241.

Karpov, K., and Cailliet, G. (1978). Feeding dynamics of *Loligo opalescens*. *Calif. Dep. Fish Game Fish Bull* 169, 45-65.

Kear, A.J., and Boyle, P.R. (1992). Loss of meal antigenicity during digestion in *Sepia officinalis* (Cephalopoda: Sepioidea). *Journal of the Marine Biological Association of the United Kingdom* 72, 543-551.

Lipiński, M. (1987). Food and feeding of *Loligo vulgaris reynaudii* from St Francis Bay, South Africa. *South African Journal of Marine Science* 5**,** 557-564.

Mangold, K., and Bidder, A. (1989). L'appareil digestif et la digestion. *Traité de Zoologie: Anatomie, Systématique, Biologie: Céphalopodes* 5**,** 321-373.

Martínez, R., López-Ripoll, E., Avila-Poveda, O.H., Santos-Ricalde, R., Mascaró, M., and Rosas, C. (2011a). Cytological ontogeny of the digestive gland in post-hatching *Octopus maya*, and cytological background of digestion in juveniles. *Aquatic Biology* 11**,** 249-261.

Martínez, R., Santos, R., Álvarez, A., Cuzon, G., Arena, L., Mascaró, M., Pascual, C., and Rosas, C. (2011b). Partial characterization of hepatopancreatic and extracellular digestive proteinases of wild and cultivated *Octopus maya*. *Aquaculture International* 19**,** 445-457.

Quintela, J., and Andrade, J.P. (2002). Diel feeding rhythms, daily ration and gastric evacuation rates of *Sepia officinalis* in the Ria Formosa lagoon (South Portugal). *Bulletin of marine science* 71**,** 665-680.

Rowe, V.L., and Mangold, K. (1975). The effect of starvation on sexual maturation in *Illex illecebrosus* (Lesueur)(Cephalopoda: Teuthoidea). *Journal of Experimental Marine Biology and Ecology* 17**,** 157-164.

Segawa, S. (1993). "Field and laboratory feeding studies of the Japanese oval squid, *Sepioteuthis lessoniana*," in *Recent Advances in Cephalopod Fisheries Biology,* eds. T. Okutani, R.K. O'dor & T. Kubodera. (Tokyo, Japan: Tokai University Press), 499-511.

Semmens, J.M. (2002). Changes in the digestive gland of the loliginid squid *Sepioteuthis lessoniana* (Lesson 1830) associated with feeding. *Journal of Experimental Marine Biology and Ecology* 274**,** 19-39.

Westermann, B., Ruth, P., Litzlbauer, H.D., Beck, I., Beuerlein, K., Schmidtberg, H., Kaleta, E.F., and Schipp, R. (2002). The digestive tract of *Nautilus pompilius* (Cephalopoda, Tetrabranchiata): an X-ray analytical and computational tomography study on the living animal. *Journal of Experimental Biology* 205**,** 1617-1624.
